# TOWARDS GAIT-BASED SCREENING FOR COGNITIVE IMPAIRMENT

**DOI:** 10.1093/geroni/igae098.4096

**Published:** 2024-12-31

**Authors:** Haley Hentnik, Christine Phillips, Abigail Stephan, Lesley Ross, Reed Gurchiek

**Affiliations:** Clemson University, Clemson, South Carolina, United States; Clemson University, Clemson, South Carolina, United States; Clemson University, Clemson, South Carolina, United States; Clemson University, Clemson, South Carolina, United States; Clemson University, Clemson, South Carolina, United States

## Abstract

While cognitive impairment is a hallmark feature of Alzheimer’s disease, it can be difficult to detect in preclinical stages. Gait metrics, like speed and variability, have been suggested to augment early detection efforts and could be used in combination with existing cognitive-based screening tools such as the modified Telephone Interview for Cognitive Status (TICS-m). However, the specificity of these gait metrics and their relationship with cognition remains unclear. This study aimed to quantify the relationship between gait and a cognitive screening assessment (TICS-m) and cognitive attention (Useful Field of View Test, UFOV) in a sample of 106 older adults (71.9 +/- 5.9 years old). Three gait metrics were significantly (p < 0.05) correlated with TICS-m including usual gait speed (r = 0.27), gait symmetry (r = 0.30), and gait smoothness (r = -0.23). These metrics were also correlated with a composite UFOV score; the strongest relationship indicated that individuals with worse attention and slower processing speed presented with a less smooth gait (r = -0.42). Interestingly, gait smoothness was more strongly related to UFOV than was TICS-m (r = -0.30). These results corroborate previous research reporting similar relationships between visual-spatial processing and gait variability in individuals with cognitive impairment. However, gait smoothness was correlated with gait speed, and thus it remains unclear if our findings are independent of this relationship. Future research should explore the specificity of gait-based digital biomarkers of cognitive impairment and their utility for early detection and screening.

